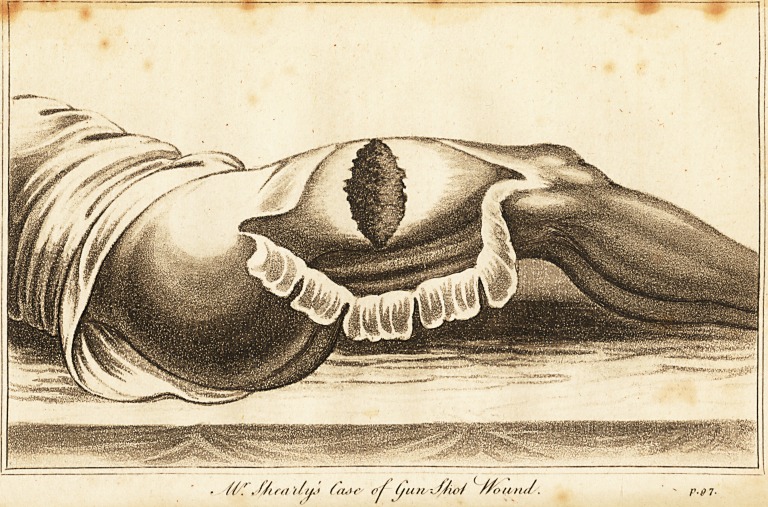# Mr. Shearly, on Gun-Shot Wounds

**Published:** 1806-02

**Authors:** W. Shearly

**Affiliations:** Member of the Royal College of Surgeons, London, Royal Naval Hospital, Deal


### Medical and Physical Journal.

VOL. XV.] FEBRUARY, 1806. [NO. 84.

*Printed for R. PHILLIPS, by W. Thorne, Red Lion Court, Fleet Street, London.*

### *To the Editors of the Medical and Physical Journal.*

GENTLEMEN,

I Must candidly confess, that I have reaped great advantages from the many valuable Communications with which your Journals have been adorned; and have therefore always thought it a duty incumbent upon me, whenever an opportunity presented, of giving a fair trial to the most important remedies, which have been recommended by your various Correspondents for the cure of divers complaints, and to make my observations upon the mode of operating in as clear and satisfactory manner as my abilities will permit. As such great panegyrics have been bestowed upon the use of Nitre in Gun-shot Wounds, by your ingenious and well informed Correspondent, Dr. Cuming; I was determined to try its powers whenever an opportunity presented. A short time had elapsed, when, on account of a severe action which was fought off the French coast, the wounded were landed at this hospital; there were a few men, who were wounded by musket balls in the upper extremities, and other parts of their bodies, in which the nitre was of evident service. But in the subsequent case, its application far surpassed my most sanguine expectations; I have therefore selected one of the most important cases, which is accompanied by a drawing, which I hope will serve to give some idea of this extensive wound, and, at the same time, demonstrate that no case could be better adapted for the experiment.

Edward Tott, a seaman, of His Majesty's ship La Fleche, was received into this hospital July 19, 1805, with a very extensive wound, which was inflicted by a cannon shot discharged from the enemy's batteries off Cape Blanc-nez. Two days had elapsed before he was presented for mission. Upon removing the bandage and dressings, a most formidable gun-shot wound presented itself; a great portion of the integuments which cover the posterior part of the thigh were carried away. The vastus externus muscle was nearly divested of its common integuments, and divided in a transverse direction; there was an opening which would readily admit two fingers to be introduced, and this opening led to the femur, which could be easily felt, covered by its periosteum. The adjacent muscles were also much injured, and the wound put on an extensive and sloughy appearance; besides the surface, which was exposed, the common integuments were undermined from the surrounding muscles to a considerable extent. The patient's pulse was full, hard, and quick, and the use of the lancet appeared to be necessary; but this was dispensed with, when we came to consider what an extensive wound we had to deal with, what an immense surface was exposed, and, moreover, the profuse suppuratiou that must necessarily come on after the sloughs had separated. A thought also struck us, that this last-mentioned process might also induce hæmorrhage, which, of course, would tend to debilitate the patient considerably and retard the cure. Fortunately for the patient, the wound was not inflicted in a vascular part, and our fears of hæmorrhage proved groundless. Scarcely had the nitre been employed four times before granulations were perceived shooting near to the wound's circumference. The nitre was applied in form of solution, viz. ℞. Pulv. nitre ℈ij. aqua mollis ℥iv. m. ft. solutio.

The whole surface of the wound was washed with this night and morning, and covered by Charpeé, the common integuments were supported with broad straps of adhesive plaster, and over all an emollient poultice was applied. Opiates and febrifuge medicines, as well as enemas, were had recourse to, and on the fifth day after this plan had been first pursued, the wound's aspect was totally changed, as not the least vestige of a slough could be perceived, and red healthy granulations were shooting up through its whole extent. In proportion as the parts returned to their wonted sensibility, so in proportion was the solution weakened, and its use was entirely laid aside on the sixth day after its first application. The irritation which the solution caused, more particularly towards the close of its use, was great for the time it lasted, but it subsided in a few minutes. The patient's constitution was now attended to, a generous generous and full diet was prescribed, and the undermined integuments, which had been detached from the surrounding muscles, had completely adhered, and cicatrization had commenced from the wound's circumference. This last-mentioned process was much accelerated by the use of lunar caustic, which was applied as near as possible to the new skin, which served as a guide in its application. The great retraction which had taken place in consequence of the transverse division of the vastus filled up amazingly fast, and the aperture which led to the femur, followed its example rapidly. The wound, about the latter end of October, was the size of half a crown, and it remained stationary till the latter end of November, when it was judged expedient to discharge him from the hospital, for the change of air, as every professional man who is at all conversant with hospital practice, must have seen how magically this change acts; and, indeed, our opinion in this case is verified, as the man has written to me, to say that his wound is perfectly cured; and the only inconvenience he complains of, is a disagreeable uneasiness in the part whenever the weather is moist. The practice of this hospital has furnished me with a great number of cases of gun-shot wounds, in which nitre proved highly beneficial, but as these are so trifling to the one I have just mentioned, that I consider the relation of them would only tend to swell unnecessarily your Journal, whose leaves might be better employed in conveying information of much more importance. I beg leave to state, that previous to seeing Dr. Cuming's remark, on the great utility of nitre in gun-shot wounds, I had witnessed its great efficacy in cleansing ulcers, degenerating from a healthy into a sloughy state. The greatest power which nitre seems to possess is, its accelerating the separation of the sloughs, and by assisting Nature in her operations. But another property which Dr. Cuming has ascribed to it, I have never detected, viz. its correcting the fœtor, which emanates, in a great degree, from this species of wounds.

I am, &c.

*Royal Naval Hospital, Deal,*
*Jan. 14, 1806.*

W. SHEARLY,
Member of the Royal College of Surgeons, London.

## Figures and Tables

**Figure f1:**